# Brain-Derived Estrogen Regulates Neurogenesis, Learning and Memory with Aging in Female Rats

**DOI:** 10.3390/biology12060760

**Published:** 2023-05-23

**Authors:** Yuanyuan Huang, Wuxiang Sun, Fujia Gao, Haoran Ma, Tao Yuan, Zixuan Liu, Huiyu Liu, Jiewei Hu, Jing Bai, Xin Zhang, Ruimin Wang

**Affiliations:** 1 Neurobiology Institute, School of Public Health, North China University of Science and Technology, Tangshan 063210, China; 15637727025@163.com (Y.H.); swx15530359711@163.com (W.S.); gaofujia1983@163.com (F.G.); mhr18732480982@163.com (H.M.); y13343808339@163.com (T.Y.); hebeitscfd6133@163.com (Z.L.); liuhuiyu1126@163.com (H.L.); jieweih2021@163.com (J.H.); baijing7858@163.com (J.B.); haohao17330544396@163.com (X.Z.); 2 School of Basic Medical Science, North China University of Science and Technology, Tangshan 063210, China

**Keywords:** aromatase, brain-derived estrogen, neurogenesis, gliogenesis, aging

## Abstract

**Simple Summary:**

This study was aimed to explore the role of brain-derived estrogen (BDE2) in hippocampal neurogenesis with aging in female rats. Our results revealed that cell differentiation was significantly declined over the middle age (14-Mon), while the differentiation of astrocytes and microglia markedly elevated and exhibited excessive activation. The newborn immature neurons clustered in hippocampal subgranular zone (SGZ) area in 1-Mon juvenile, then sharply dropped thereafter and the number of neural stem cells declined over 14-Mon age. Female forebrain neuronal aromatase knockout (FBN-ARO-KO) rats showed declined neurogenesis in dentate gyrus (DG) region at 1, 6 and 18-Mon ages, compared to WT controls. In addition, letrozole suppressed neurogenesis at 1-Mon age. On the contrary, astrogenesis was elevated over middle age and FBN-ARO-KO promoted the differentiation and activation of astrocytes and microglia in the DG region. FBN-ARO-KO rats also displayed decreased levels of CREB-BDNF signal and cognitive-related proteins, as well as impaired spatial learning and memory in juvenile (1 Mon) and adulthood (6 Mon). Our results suggest that long-term shortage of aromatase-BDE2 signaling may accelerate brain inflammation by increase local gliogenesis and activation, and that BDE2 plays a key role for the maintaining of hippocampal neurogenesis and cognitive function.

**Abstract:**

Although 17β-estradiol (E2) can be locally synthesized in the brain, whether and how brain-derived E2 (BDE2) impacts neurogenesis with aging is largely unclear. In this study, we examined the hippocampal neural stem cells, neurogenesis, and gliogenesis of 1, 3, 6, 14, and 18-month (Mon) female rats. Female forebrain neuronal *aromatase* knockout (FBN-ARO-KO) rats and letrozole-treated rats were also employed. We demonstraed that (1) the number of neural stem cells declined over 14-Mon age, and the differentiation of astrocytes and microglia markedly elevated and exhibited excessive activation. KO rats showed declines in astrocyte A2 subtype and elevation in A1 subtype at 18 Mon; (2) neurogenesis sharply dropped from 1-Mon age; (3) KO suppressed dentate gyrus (DG) neurogenesis at 1, 6 and 18 Mon. Additionally, KO and letrozole treatment led to declined neurogenesis at 1-Mon age, compared to age-matched WT controls; (4) FBN-ARO-KO inhibited CREB-BDNF activation, and decreased protein levels of neurofilament, spinophilin and PSD95. Notably, hippocampal-dependent spatial learning and memory was impaired in juvenile (1 Mon) and adulthood (6 Mon) KO rats. Taken together, we demonstrated that BDE2 plays a pivotal role for hippocampal neurogenesis, as well as learning and memory during female aging, especially in juvenile and middle age.

## 1. Introduction

Neurogenesis refers to the process of generating new functional neurons from neural stem cells (NSCs), which present in the brain throughout the lifespan. All the events are based on a series of cellular processes, such as proliferation, differentiation, migration, maturation, and ultimately synaptic integration into existing neural circuits [1,2]. NSCs mainly originate from the subventricular zone (SVZ) and the subgranular zone (SGZ) of the hippocampal dentate gyrus (DG). NSCs from SGZ can migrate a short distance and differentiate into the immature neuron and then as excitatory granule cells to integrate into DG [1,3]. In line with this, newborn neurons play crucial roles in modulating hippocampal function, such as learning and memory [4], social behavior [5] and pattern separation [6].

The process of neurogenesis is affected by the brain microenvironment, such as gliogenesis, the extracellular matrix and secreted hormones. Unlike neurogenesis, gliogenesis including proliferation of astrocyte, microglia, and oligodendrocyte cells occurs more prevalently in the adult brain, which secret cytokines and chemokines, by which to modulate neurogenesis [7,8]. Therefore, the imbalance between neurogenesis and gliogenesis may trigger central neural system (CNS) disorders. Indeed, more and more evidence indicates that neurogenetic damage is the molecular basis of various diseases such as juvenile autism, neurodevelopmental disorders [9], adult schizophrenia, anxiety, depression [10,11,12], and senile neurodegenerative diseases [13]. Conversely, brain injury stimulation can also lead to abnormal gliogenesis, resulting in improper migration, reduce survival of newborn neurons, and hampering the repair of damaged-brain [14,15,16]. Hence, revealing the rules of neurogenesis and gliogenesis and the impacts during aging will help to understand the molecular mechanisms of CNS diseases. 

Increasing evidence indicates that CNS diseases such as depression, and Alzheimer’s disease have a higher incidence in females than the same age male, suggesting a crucial role of sex hormones [17]. 17β-estradiol (E2) is a steroid hormone that plays neurotrophic and extensive neuroprotective roles in the brain, including antioxidant stress, anti-inflammatory damage, increasing synaptic plasticity, and improving cognition [18]. E2 replacement rapidly upregulates cell proliferation in the DG of adult female rats [19]. Furthermore, E2-treatment also induces neurogenesis and promotes hippocampal-dependent learning and memory across the lifespan [20]. In female CNS, there are two main sources of E2: (a) circulation synthesized by ovaries, and (b) locally catalyzed and produced by aromatase from substrate testosterone [21]. Some studies have reported that aromatase activity in the brain of healthy elderly women is increased, with brain-derived estrogen (BDE2) being six times higher than circulating E2 [22,23]. Our recent study confirms that in 18-month (Mon) old female rats, E2 level in the hippocampus is significantly higher than that in the serum [24]. Conditional knockout *aromatase* gene in forebrain neurons leads to cognitive dysfunction in rats and mice [25]. Therefore, it is reasonable that BDE2, rather than circulating estrogen, is a more direct regulator in maintaining brain functions [26]. However, largely is unclear whether BDE2 participates in the mediation of neurogenesis during pre-sexual maturation and postmenopausal periods, when circulating estrogen levels are extremely low in females. 

In this study, we used the female rats aged at 1, 3, 6, 14, and 18 Mon, which represents the natural aging process in juvenile, emerging adulthood, young adulthood, middle age (perimenopause), and postmenopause (18 Mon), respectively [27]. To elucidate the effects of BDE2 on neurogenesis with aging, we generated *aromatase* knockout rats in forebrain neurons (FBN-ARO-KO, KO), and 1-Mon (intact), 6-Mon (ovariectomized, OVX) and 18-Mon (intact) KO females rats were used in this study. We aimed to investigate (1) the alternations in differentiation of NSCs in the hippocampus DG region with natural aging; (2) the key roles of aromatase-BDE2 signaling in mediating neurogenesis, gliogenesis, and hippocampal-dependent cognitive function using the KO rats; (3) the effects of letrozole on BDE2 and neurogenesis using 1-Mon female rats. Our study could provide new insight into the complexity of the aging process an important clue of BDE2 for preventing CNS diseases.

## 2. Materials and Methods

### 2.1. Antibodies

The following primary antibodies were used in this study: Estradiol (BioGenex, San Francisco, CA, USA #AR038), NeuN (Millipore Biotechnology, Massachusetts American #NG1857584), DCX (Santa, TX, USA #sc8066), SOX2 (Abcam, Cambridge, UK #ab97959), GFAP (Abcam, #ab53554), Iba1 (Abcam, #ab175076), MAP2 (Santa, #sc8066), MBP2 (Proteintech, Chicago, IL, #NO.10458-1-AP), GAPDH (Proteintech, #NO.60004-1-1g), p-CREB (Cell-signaling, Boston, MA, USA #9198s), CREB (Abclonal, Wuhan, China #56653), BDNF (Arigo, Shanghai, China #56653), PSD95 (Abcam, #ab1825), Neurofilament (Abcam, #ab7794), Spinophilin (Abcam, #ab13359) and SNAP25 (Abcam, #ab5666).

### 2.2. Experimental Design

Adult female Sprague-Dawley rats were from Beijing Huafukang Biotechnology Co., Ltd., China [Animal License No.: SCXK (Beijing) 2020-0004], and were divided into five groups: 1-month-, 3-month-, 6-month-, 14-month-, and 18-month-age rats. To elucidate the effects of BDE2 on differentiation and neurogenesis, we generated a rat model of *aromatase* gene knockout specific in forebrain neurons (FBN-ARO-KO, KO) using Cre-lox P technology by Beijing Biopsy Pharmaceutical Technology Co., Ltd., China [animal license number: EGE-LJL-101 cKO-20190218], and 1, 6, and 18-month-age KO female rats and age-matched WT rats were used in this study. To rule out the effects of circulating E2, 6-months old female rats underwent either bilateral ovariectomy (OVX) under anesthetization with isoflurane. 

It is reported that NSC in the hippocampal DG region are sharply decreased during the first year after born [28]. Additionally, except for resting neurons, aromatase is also expressed in astrocytes, and even upregulation in aromatase levels in response to stress impairments such as ischemia [29,30] and traumatic brain injury [31,32]. Thus, in order to inhibit total aromatase activity, 3-week-WT rats were selected and intragastrically administrated with aromatase inhibitor letrozole (Sigma-Aldrich, Shanghai, China 10 mg/kg) once a day for 5 days, according to previous dose [33,34]. All tissue samples of experimental animals were collected in the morning. All animal protocols were approved by the Animal Ethics Committee of North China University of Science and Technology. The rats were fed freely at constant temperature (22~24 °C) with the circadian rhythm (12 h light/12 h dark). The experiment was conducted in accordance with the guidelines formulated by the Animal Ethics Committee (AEC) and the National Natural Science Foundation Committee, and all appropriate measures were taken to minimize pain in the animals.

### 2.3. Immunofluorescence Staining and Confocal Microscopy

Immunofluorescence staining (IF) was performed as previously described in our study [35]. In brief, the experimental rats were transcardially perfused with 0.9% saline under deep anesthesia with 2% isoflurane, and the brain was imminently removed from the skull and postfixed in 4% paraformaldehyde in 0.1 M PB for 4 °C overnight. After dehydration in 30% sucrose, the tissues were embedded in an optimal cutting temperature (OCT) compound. Serial coronal sections (25 µm, 2.5–4.5 mm posterior from bregma) were made by a frozen slicer (Leica RM2135, Germany) and stored at −20 °C in an antifreeze solution until using. Brain sections were washed 3 times for 10 min each in 0.1 M PBS and then blocked in 10% normal donkey serum in 0.1% Triton X-100-PBS for 1 h at room temperature, followed by 48 h incubation in primary antibodies anti-NeuN (1:300), anti-DCX (1:100), anti-GFAP (1:1000), anti-SOX2 (1:500), anti-Iba1 (1:1000) at 4 °C. Followed by washing in 0.1% Triton X-100-PBS, the brain sections were incubated with secondary antibodies (Alexa Fluor donkey anti-mouse/goat/rabbit 488/594/647 nm, Thermo Fisher) at room temperature for 1.5 h and then washes four times using washing buffer (0.1% Triton X-100-PBS) for 20 min each. The sections were then mounted on slides, and coverslipped with DAPI mounting medium (Lot ZA0210, Vector Laboratories, Inc. Burlingame, CA, USA). Images were acquired on a confocal laser microscope (Andor Dragonfly) using a 10× or 40× objective lens with the image size set at 1024 × 1024 pixels. Co-labeled cells of double immunofluorescence staining or fluorescence intensity were quantative evaluated by ImagJ Fiji software. 

### 2.4. Cell Quantifications and Morphometric Measurement

To build 3D images of glial cells in the hippocampal DG regions, confocal single plane images and Z stacks with a step size of 1.0 μm were taken under a 40× objective lens of a confocal laser microscope. We processed the immunofluorescence staining images of GFAP or Iba1 into 3D constructions and assessed volume of glial cells using Imaris 9.5 software (Bitplane AG) [36]. At least 4~7 randomly selected sections per animal were used for immunostaining, and the typical image was selected for presentation. 

The SOX2/GFAP immunofluorescence sections were also digitalized at 20× magnification utilizing a TissueFaxs Plus System. Regions of interested were drawn manually using the zooming and mark-up tools included in the TissueFAXS viewer [37]. The intensity of signals localized onto the sections was then evaluated.

### 2.5. Preparation of Hippocampal Samples and Western Blot Analysis

At designed time points, the rats were anesthetized and transcardially perfused with cold saline. The rat brain was quickly decapitated, and the hippocampus was separated on ice and stored at −80 °C immediately. Protein extractions were prepared with lysis buffer (50 mM Tris-HCl, pH 7.4, 150 mM NaCl, 5 mM EDTA, 0.5% NP-40, 0.1% Triton X-100, 0.1% SDS, 1 mM PMSF, 1× protein inhibitor mix Complete Mini) and then centrifuged at 10,000× *g* for 10 min at 4 °C. Protein concentrations were examined and standardized using a BCA protein assay kit (Pierce, Rockford, IL, USA). The protein sample (20 µg per lane) was separated by 10% SDS-PAGE and transferred to a polyvinylidene fluoride (PVDF) membrane. The membranes were blocked with 3% BSA for 1 h and then incubated overnight at 4 °C with the primary antibodies. Membranes were washed with TBST and incubated with the secondary antibody for 1 h. The signal was visualized by enhanced chemiluminescence (ECL prime; Amersham Biosciences, Piscataway, NJ, USA). The protein levels were quantitatively analyzed using Image Lab software version 5.2.1 (Bio-Rad). The protein levels were normalized by those of GPADH, a loading control.

### 2.6. Quantitative RT-PCR Analysis

To measure mRNA expression of astrocyte A1/A2 markers, total RNA was isolated from the hippocampal tissue, which was collected on ice and stored at −80 °C immediately. RNA was extracted with TRIzol reagent (Cat# 15596-026, Life Technologies, Carlsbad, CA, USA) according to the manufacturer’s instructions. The purified RNA was then reverse-transcribed into cDNA using the Reverse Transcription System (RR047A, TaKaRa PrimeScript™ RT reagent Kit). Quantitative RT-PCR analysis of the mRNA levels of target genes was performed using the TB Green Premix Ex Taq kit (RR820A, TAKARA). The real-time PCR program steps were: 95 °C for 5 min, 45 cycles at 95 °C for 5 s, 60 °C for 5 s, and 72 °C for 10 s, followed by 72 °C for 1 min. The primers of targeting genes used in the study were listed in the Table 1.

### 2.7. Behavioral Assessments

Barnes Maze Test Barnes maze test was performed as previously described [38]. Briefly, the Barnes maze consists of a large circular platform containing 18 holes on the outer edge and elevated approximately 120 cm above the floor. A black escape box (20 × 15 × 12 cm^3^) is hidden under one of these holes, in which the animal can hide, while the other holes are left open to the floor. An overhead light (500 W, 1000 lux) is shining down on the platform surface, and a loud (60 dB) repetitive tone is playing during the test. The Barnes maze task included three days of twice-daily 3 min training trials, followed by a 90 s probe trial on the fourth day. During the training trail, the escape hole position was fixed for all days of the task. During training trials, the researcher allowed the rats up to 3 min to enter the black escape box. The time taken for the rat to locate and enter the escape box is defined as the escape latency. If the animals failed to identify the escape hole within 3 min, they were gently guided towards the escape hole, remained in the box for 30 s to habituate, and were given a score of 3 min. On day 4 of the probe trial, the escape box was removed, and the corresponding hole was blocked. The time spent in the target quadrant, where the target box is located, was recorded and analyzed by ANY-maze video tracking software (Stoelting; Wood Dale, IL, USA). Between each trial, both the maze platform and escape hole were thoroughly cleaned with 70% EtOH to remove any scent cues that might affect performance in subsequent trials.

Open field test the open field test is used to assess locomotor activity and anxiety-like behavior [39]. The test was conducted for 5 min in a dimly lit room, as described previously [40]. The apparatus consisted of a large arena measuring 100 × 100 × 50 cm^3^ (L × W × H). The arena was made of black high-density polyethylene panels that were fastened together and placed on a plastic bottom plate. The rats were traced by an overhead video camera controlled by the ANY-maze video tracking software. At the beginning of the test, a mouse was placed at center of the box. During a 5-min period, the rearing times, the grooming time, as well as time spent in a delineated center zone (50 × 50 cm^2^) were recorded.

### 2.8. Statistical Analysis

All experiments in the current study were performed on age-matched female WT and FBN-ARO-KO rats. All the data were analyzed with SigmaStat 3.5 software. The comparison between the two groups was conducted by the *t* test. One-way analysis of variance (ANOVA) tests with Student-Newman-Keuls post hoc tests were performed to determine differences among independent groups. Data were expressed as mean ± SEM. The statistical figures were generated by GraphPad 9 software.

## 3. Results

### 3.1. Decline in the Number of Neural Stem Cells over Middle Age, and Sharp Drop in Neurogenesis after Birth in the Hippocampal DG Region of Female Rats

**Figure 1:**
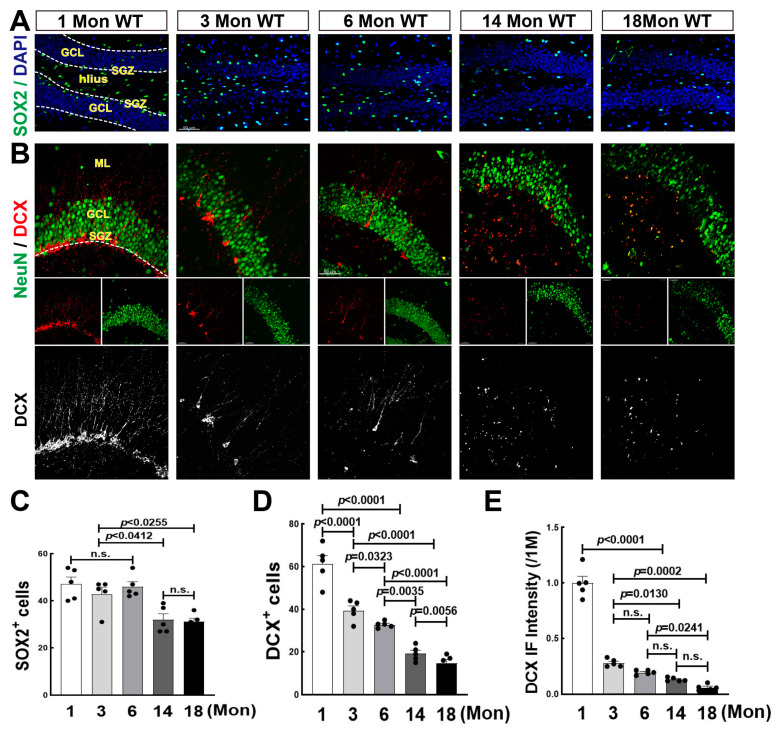
Changes of Neural Stem Cell and Neurogenesis in the Hippocampal DG Region of Female Rats. (**A**) Representative images of SOX2 immunofluorescence staining (green). Hippocampal coronary sections were from female 1-, 3-, 6-, 14- and 18-Mon old rats. (**B**) Representative images of double immunofluorescence staining for DCX (red, a marker of immature neuron) and NeuN (green, a marker of the mature neuron) in the indicated groups. DCX+ staining were highted in the bottom panel. (**C**) Quantitative analysis of SOX2+ cells by Fuji software. DAPI (blue) was used to counterstain nuclei. (**D**,**E**) Quantitative analysis of DCX+ cells and DCX fluorescence intensity by Fuji software. Data were expressed as means ± SEM, n = 5 for each group. Scale bar, 50 μm, magnification 20× in Figure 1A, 40× in Figure 1B.

Increasing evidence reveals that neurogenesis is constantly occurring throughout life, while some debate that it is undetectable after adulthood in humans [28,41]. We then performed immunofluorescence staining for SOX2, a marker for NSCs [42] in SGZ subregion of hippocampal DG during aging, one of the NSCs niches where NSCs differentiate into immature neurons [28]. The representative images of SOX2 staining and the quantitative analysis were shown in Figure 1A,C. The majority of SOX2+ cells (green) were localized along the border of SGZ at 1 Mon, and dispersed throughout DG region including GCL and hilus over 3 Mon. We found that the numbers of SOX2+ cells in 1, 3, and 6-Mon rats had no significant difference, while markedly declined at 14 and 18 Mon of age. We next conducted double immunofluorescence staining for DCX (red) and NeuN (green), markers of immature and mature neurons, respectively. As expected, the number of DCX+ cells dramatically decreased after 1 Mon age (Figure 1B,D). A few typical DCX+ cell bodies with long dendrites were seen in the 3-Mon and 6-Mon rats, and few cellular bodies of DCX+ staining could be seen at 14 Mon and 18 Mon. In the 1-Mon rats, DCX+ cells ranked alongside SGZ subregion with abundant long dendrites. At 3 Mon and 6 Mon, a few single DCX+ cells were co-labeled with NeuN (yellow) in the innermost layer of GCL (Figure 1B). Fluorescence intensity of DCX+ staining sharply declined after 1 Mon and gradually dropped with aging (Figure 1B,E). The results suggest that neurogenesis occurs throughout rat life, postnatal neurogenesis is active, and then significantly declines over middle age. 

### 3.2. FBN-ARO-KO Suppressed NSC Differentiation and Neurogenesis in the Hippocampal DG Region

**Figure 2:**
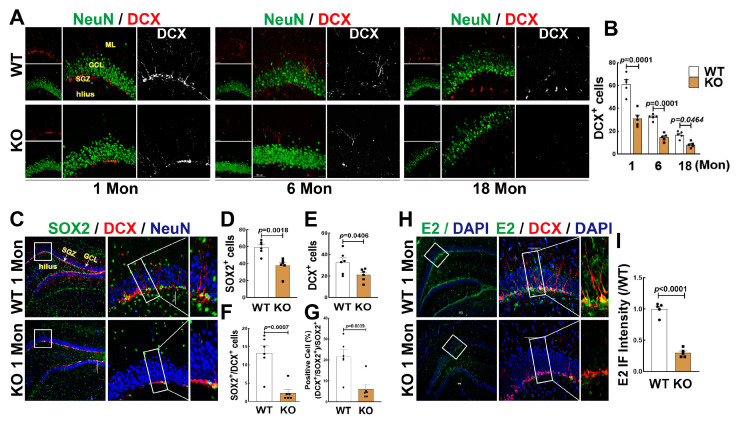
Effects of Aromatase on Neurogenesis in the hippocampal DG region of female rats. (**A**,**B**) Representative images of double immunofluorescence staining for DCX (red) and NeuN (green), and quantitative analysis of DCX+ cells in the hippocampal DG region of WT or FBN-ARO-KO rats at 1-, 6-, and 18 months old. (**C**) Representative images of triple immunofluorescence staining for SOX2 (green), DCX (red), and NeuN (blue) using the brain sections from 1-Mon rats. Quantitative analyses of SOX2+ (**D**), DCX+ (**E**), and co-labeled SOX2+ with DCX+ cells (yellow, (**F**)), as well as the ratio percentage of DCX+/SOX2+ to SOX2+ cells (**G**). (**H**,**I**) Immunofluorescence staining for E2 and quantitative analysis of E2 fluorescence intensity in the hippocampal DG region, and DAPI was used to conterstain nuclei (blue). Analyses were expressed as means ± SEM, n = 5–6 for each group. Scale bar, 50 μm; magnification, 40×.

FBN-ARO-KO rats and age-matched WT rats were used to determine the changes of neurogenesis in the hippocampal DG region. As shown in Figure 2A,B, FBN-ARO-KO significantly decreased DCX+ cells (red) in the 1, 6 and 18 Mon aged rats, compared to age-matched WT animals. To evaluate the effect of BDE2 on the ratio of neurogenesis, we performed triple immunofluorescence staining for SOX2 (green), DCX (red), and NeuN (blue). As shown in Figure 2C,D–G, KO rats exhibited fewer numbers of SOX2+ and DCX+ cells compared with WT controls. Additionally, the number of DCX+/SOX2+ was significantly decreased compared to the WT controls (Figure 2C,F). The percentage ratio of DCX+/SOX2+ cells to SOX2+ cells in WT and KO rats were 21.495 ± 3.475 and 6.096 ± 2.241, respectively (Figure 2C,G). Finally, we performed immunofluorescence for E2 (green) and DCX+ (red), we were surprised to find that E2 strongly displayed in SGZ area, highly co-labeling with DCX+ cells in the 1 Mon WT rats. The fluorescence intensity of E2 was significantly decreased in 1 Mon KO rats compared to WT group (Figure 2H,I). These results suggest that BDE2 may contribute to NSC formation and neurogenesis.

### 3.3. Knockout Aromatase in Forebrain Neurons Leads to Hippocampal Neuronal Impairment and Cognitive Defect

**Figure 3:**
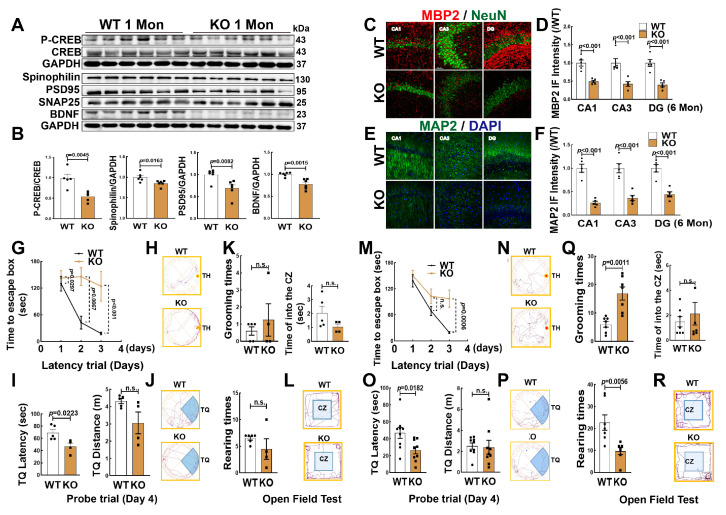
FBN-ARO-KO Impaired Hippocampal Neurons and Cognitive Function. (**A**,**B**) Western blot analysis for the indicated proteins in the hippocampus of 1-Mon KO and WT female rats. Data analyses of were expressed as ratios to total CREB or GAPDH (a loading control). Data were expressed as means ± SEM, n = 5–6 for each group. (**B**–**D**) Representative images of immunofluorescence staining for MBP2 (red, a marker of myelin sheath) and NeuN (green, a marker of surviving neuron) and quantity of MBP2 fluorescent intensity in the hippocampal subregions (CA1, CA3 and DG). (**E**,**F**) Representative images of immunofluorescence staining for MAP2 (green, a dendritic marker) and quantity of MAP2 fluorescent intensity in the hippocampal subregions. DAPI was used to conterstain nuclei (blue). Scale bar, 50 µm; magnification, 40×; Data are shown as means ± SEM. n = 5 in each group. (**G**,**I**) 1 Mon intact female rats and (**M**,**O**) 6 Mon OVX rats. The latency trial (**D**,**G**,**M**) and probe trail (**I**,**O**). Representative tracking plots in the escape latency (**H**,**N**) and probe trail **(J**,**P**). Open field test both in 1 Mon (**K**,**Q**) and 6 Mon rats were performed with grooming times, total grooming time, rearing times, total rearing time, as well as the time of entering the center zone (CZ). **(L**,**R)** Representative tracking plots of open field test. Data are shown as means ± SEM. n = 6–8 in each group. n.s., no significance. The full WB figures refer to the Supplementary Materials Figure S1.

We next determined whether FBN-ARO-KO impaired hippocampal neurons and cognitive function in juvenile (1 Mon) and adolescent (6 Mon) female rats. Western blot analysis revealed that the protein expression of spinophilin, PSD95, BDNF and p-CREB/CREB were significantly decreased in the hippocampus of 1-Mon KO rats, compared to the WT controls (Figure 3A,B). In addition, we selected brain coronal sections from 6-Mon ovariectomized (OVX) rats and examined MBP2 and MAP2 proteins. As shown in Figure 3, double immunofluorescence staining for NeuN (red) and MBP2 (green) revealed that the fluorescent intensity of MBP2 (Figure 3C,D) and MAP2 significantly decreased in the hippocampal subregions (CA1, CA3, DG) in KO rats, compared to age-matched WT rats (Figure 3E,F). 

Next, we subjected 1-Mon (intact) and 6-Mon rats (OVX) to the Barnes maze task for the evaluation of hippocampal-dependent spatial learning and memory. The results revealed that in the two time points, KO rats of both ages displayed a significant attenuation in spatial learning and memory, as evidenced by a longer latency time to find the target hole (TH) on the third day of the latency trial (Figure 3G,H) and a shorter explore time in the target quadrant (TQ) of probe trial than that in WT animals (Figure 3I,J). The exploration distance (TQ distance) was similar between the two groups. The open-field test is widely used to assess locomotor activity and anxiety in rodents [25]. The results revealed that both 1-Mon KO and the age-matched WT rats exhibited similar rearing/grooming times and traveling distance in the central zone (Figure 3K,L). However, at the 6 Mon time point, KO rats exhibited more grooming times and less rearing times than that of WT rats, while there was no difference in the time spent in the central zone between the two groups (Figure 3Q,R). Figure 3 M–P are the representative traces in latency and probe trails, respectively. Together, these results suggest that brain aromatase is an essential factor in maintaining neuronal health and hippocampal-dependent cognitive function.

### 3.4. Letrozole Suppressed Neurogenesis and Cognitive Related Proteins in DG of 1-Mon Female Rats

**Figure 4:**
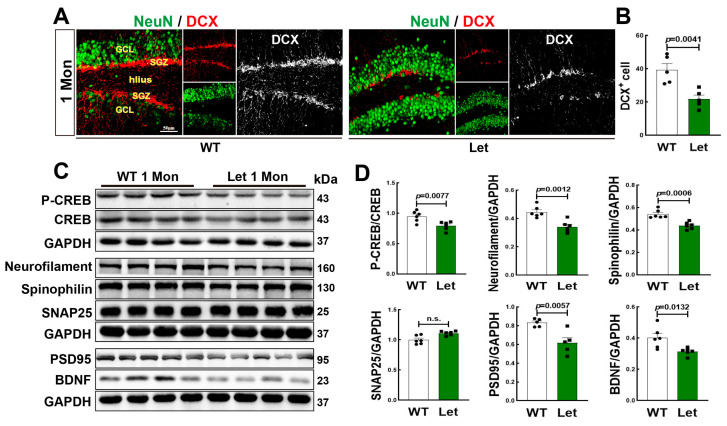
Letrozole Inhibited Neurogenesis and Neuroprotection in the Hippocampal DG Region of 1-Mon Female Rats. (**A**,**B**) Confocal microscope for DCX+ (red), NeuN (green) and data analysis in the hippocampal DG region of control (WT) and letrozole-treated rats. (**C**) Western blot analyses of p-CREB/CREB, neurofilament, spinophilin, SNAP25, PSD95 an BDNF in the hippocampus. Quantification of Western blot data shown in (**D**). n = 5–6 for each group. Let: letrozole. The full WB figures refer to the Supplementary Materials Figure S2.

We administrated letrozole to inhibit aromatase activity of 1-Mon female rat. Double immunofluorescence staining for DCX+ (red) and NeuN+ (green) showed a significant decrease in DCX+ cells in DG region of 1-Mon old letrozole-treated rats, compared to the controls (Figure 4A,B). Neurofilaments belong to the cytoskeletal intermediate filament proteins as biomarkers in neurological disorders [43]. Therefore, we determined cognitive related proteins, and Western blot analysis revealed that except for SNAP25, protein levels of neurofilament, spinophilin, PSD95, p-CREB/CREB, and BDNF were significantly decreased in the hippocampus of letrozole-treated rats, compared to control animals (Figure 4C,D). Taken together, the findings confirm the crucial role of aromatase in neurogenesis and cognitive related proteins.

### 3.5. Astrogenesis Was Elevated in the Hippocampal DG Region over Middle Age in Female Rats

**Figure 5:**
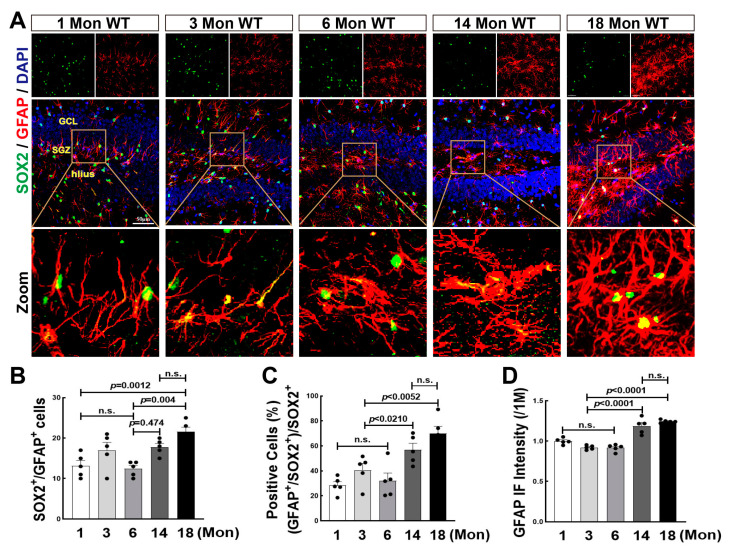
Astrocyte Proliferation and Activation Were Enhanced over Middle-age of Life in the Hippocampal DG Region of Female Rats. (**A**) Representative photographs of immunofluorescence staining of SOX2 (green) and GFAP (red), and DAPI (blue) was used to counterstain nuclei. Quantitative analysis ofSOX2+/GFAP+ cells (**B**), the percentage ratio of astrocyte differentiation (SOX2+/GFAP+) in total SOX2+ cells (**C**), and fluorescent intensity of GFAP (**D**). Data were expressed as means ± SEM, n = 5. Scale bar, 50 μm; magnification, 40×.

NSCs can also differentiate into glial cells, such as astrocytes and microglia, and further reversely affects NSCs differentiation as well as newborn neuronal survival by secreting cytokines and chemokines [44]. Previous studies from ours and others demonstrate that aromatase is highly expressed in astrocytes in response to ischemic stroke and traumatic brain injury [45,46]. We recently found that lack of aromatase-BDE2 resulted in cognitive dysfunction in 18-Mon female rats. In order to reveal the role of BDE2 in neurogenesis during the natural aging of female rats, we then performed immunofluorescence staining for SOX2 (green) and GFAP (red) in 1, 3, 6, 14, and 18-Mon female rats. As shown in Figure 5A, in 1-Mon and 3-Mon rats, astrocytes display elongated processes and smaller cell bodies, however, astrocytes were gradually activated from 6 Mon to 18 Mon, evidenced by larger volume and shorter processes. Quantitative analyses revealed that the number of GFAP+/SOX2+ cells was significantly increased in 14-Mon and 18 -Mon groups. The percentage ratio of SOX2+/GFAP+ cells to SOX2+ cells was markedly increased, compared to the 1, 3 and 6-Mon animals (Figure 5A–C). GFAP fluorescence intensity was significantly enhanced over middle age, indicating hyper-activation of astrocytes (Figure 5A,D).

### 3.6. FBN-ARO-KO Promoted Astrogenesis in the Hippocampus and the Changes of A1/A2 Reactive Astrocytes in 18-Mon Female Rats 

**Figure 6:**
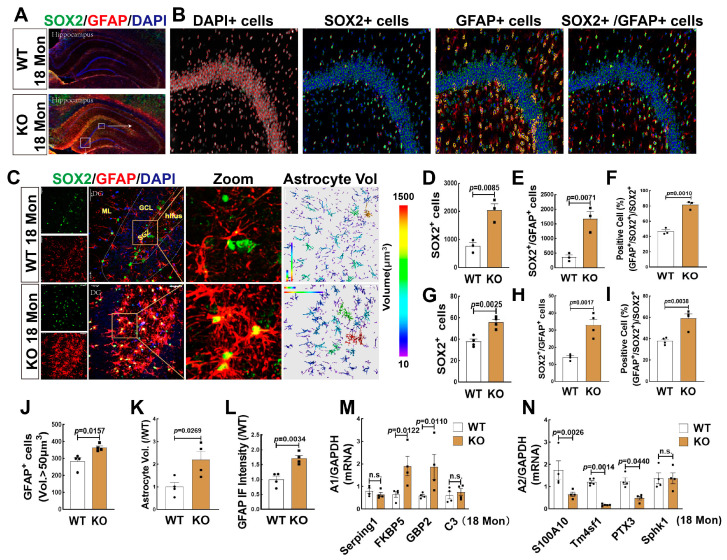
FBN-ARO-KO Promoted NSCs differentiation to Neurotoxic Astrocytes in the Hippocampal DG Region of 18 Mon Female Rats. (**A**,**B**) Representative hippocampal sections from 18-Mon WT and KO rats were subjected to double immunofluorescence staining for SOX2 (green) and GFAP (red). Quantification was performed by counting the numbers of SOX2+ (**D**), and SOX2+/GFAP+ cells (**E**) by TissueFAXS Spectra Systems and StrataQuest analysis software. The percentage of SOX+/GFAP+ ratio to total SOX2+ cells serves as the differentiation rate to astrocytes (**F**). (**C**) Hippocampal DG images of immunofluorescence staining for SOX2 with GFAP and quantitative analyses showed the number of SOX2+ cells (green, **G**), and GFAP+/SOX2+ cells (yellow, **H**). The percentage changes of SOX2+/GFAP+ ratio to total SOX2+ cells (**I**). Astrocytic volume (**J**,**K**) and fluorescence intensity (**L**) of GFAP+ staining were evaluated. (**M**,**N**) A1/A2 astrocyte gene expression in 18 Mon WT and KO rats was verified using RT-qPCR (n = 4). Data were shown as mean ± SEM, n = 3–4, magnification 20× in Figure 6A; n = 4, magnification 40× in Figure 6B,C Z-stack 1 µm (total 20 µm).

We then detected the effect of FBN-ARO-KO on astrogenesis in female rats, as it may reflect changes in the microenvironment of newborn neurons with aging. We first conducted immunofluorescence staining for SOX2 and GFAP in WT and KO rats at 18-Mon age, which refers to the late stage of middle-age of life [27]. Figure 6A,B showed representative 3D reconstructed images of hippocampal panoramic and amplified images of DG region, which illustrated the process for quantitative analyses of SOX2+ (green), GFAP+ (red), and SOX2+/GFAP+ cells by TissueFAXS Spectra Systems and StrataQuest analysis software. Intriguingly, the results revealed that compared to the WT group, KO rats exhibited significant increases in both SOX2+ (Figure 6D) and co-labeling cells of SOX2+ with GFAP+ (Figure 6E). The percentage of SOX2+/GFAP+ cells ratio to SOX2+ cells was markedly increased in KO rats, compared to WT controls (Figure 6F). Furthermore, we evaluated astrogenesis in the hippocampal DG region (Figure 6C), and the results showed that the numbers of SOX2+ cells (green) (Figure 6G), the co-labeled cells of SOX2+and GFAP+ (yellow) (Figure 6H), and the percentage of SOX2+/GFAP+ cells ratio to SOX2+ cells (Figure 6I) were significantly increased in the KO group, compared to WT rats. Astrocyte immunoreactivity was evaluated by analyzing astrocyte volume and fluorescence intensity. Data analysis showed that both the number of astrocytes with a volume greater than 50 µm^3^ (Figure 6J) and the total astrocyte volume (Figure 6K) were significantly enhanced in the KO group, compared to WT rats. We also demonstrated that the fluorescence intensity of GFAP+ staining in the KO group was markedly elevated compared to the WT control (Figure 6L). 

Astrocytes are currently divided into two subtypes A1 and A2, the former is believed to exert proinflammatory injury, while the latter refers to pro-survival function [47,48]. We thus detected some markers of A1 and A2 astrocytes by RT-qPCR, and the results showed that mRNA levels of A1 markers FKBP5 and GBP2 were significantly increased, while mRNA levels of A2 markers S100A10, Tm4sf1, and PTX3 were significantly decreased in KO group, compared to the WT control. There were no significant changes in mRNA levels of A1 markers Serping1, C3, and A2 marker Sphk1 (Figure 6M,N). These findings indicated that lacking of brain aromatase in forebrain neurons induced astrogenesis and A1 neurotoxic astrocytes, which might result in a neuro-inflammatory environment that is not conducive to the survival of newborn neurons.

### 3.7. Effects of Aging and FBN-ARO-KO on Microglia Differentiation and Activation in the Hippocampal DG Region of 18-Mon Female Rats

**Figure 7:**
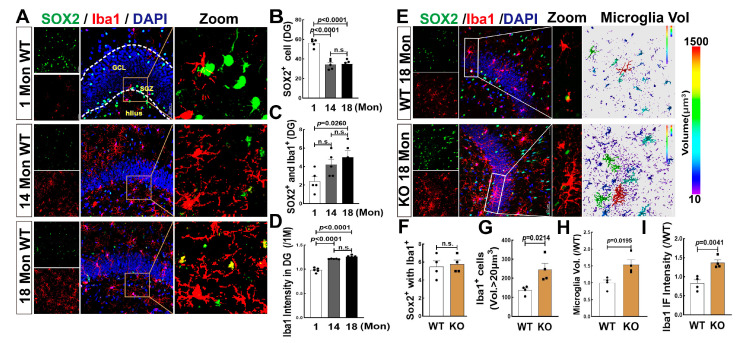
Microglial Cell Proliferation is Increased in Aged Female Rats and FBN-ARO-KO Induces Inflammatory Impairment in the Hippocampal DG Region. (**A**) Representative microscopy images of Iba1 (red, a marker of microglia) and SOX2 (green) in the hippocampal DG region of 1-Mon, 14-Mon and 18-Mon female rats. (**B**,**C**) Co-localization of Iba1+ with SOX2+ (yellow) indicates microglia cells differentiated from NSCs. (**D**) Quantitative analysis of Iba1 intensity (ratio to 1 Mon) was performed using Fuji software. (**E**) Immunofluorescence staining for Iba1 (red) and SOX2 (green), showing no significant change in co-localized cells of Iba1+ with SOX2+ (**F**), compared to the WT group. 3D analysis by Imaris 9.5 shows the increased volume (**G**,**H**) and fluorescence intensity (**I**) of Iba1+ cells in KO rats, indicating microglia over-activation compared to WT animals. DAPI was used to counterstain nuclei. Data were expressed as mean ± SEM, n = 4–5, magnification 40×, scale bar 50 µm, Z-stack 1 µm (total 20 µm).

We also evaluated the genesis of microglia in the hippocampal DG region using Iba1 (red) and SOX2 (green) labeling and 3D evaluation in 1, 14, and 18 Mon female rats. Similar to astrocytes, we observed a significant increase in double-positive cells (SOX2+ with Iba1 +) in 18 Mon rats, compared to 1-Mon and-14 Mon rats, although SOX2+ cells markedly declined after 14 months of age (Figure 7A–C). Iba1 fluorescence intensity was significantly increased in 14 and 18-Mon rats, compared to 1-Mon group (Figure 7A,D). Finally, we observed excessive activation but not differentiation of microglia in the hippocampal DG region of 18-Mon KO rats compared to age-matched WT rats, as evidenced by hypertrophic and amoeboid-shaped Iba1+ cells in the KO rats, while no change in SOX2+/Iba1+ double-positive cells of between the two groups (Figure 7B,F). Iba1+ cells with a volume greater than 20 µm^3^ (Figure 7B,G) and the total astrocyte volume (Figure 7B,H) were significantly enhanced in the KO group, compared to WT rats. The fluorescence intensity of Iba1+ staining in the KO group was markedly elevated compared to the WT control (Figure 7B,I). Taken together, these findings suggest that the aromatase ablation in forebrain neurons may induce inflammatory injury via microglia activation in the hippocampal DG region over middle age in female rats.

## 4. Discussion

The high plasticity of the adult hippocampus is closely related to neurogenesis, and its structure and function are extremely sensitive to estrogen. Estrogen participates in the regulation of hippocampus-dependent learning and memory throughout the female life cycle, including the physiological cycle, pregnancy, lactation, perimenopause, and post-menopause [49,50,51]. However, most current studies have focused on the role and regulation of ovarian-derived estrogen [52,53]. In this study, we aimed to uncover the regulatory effect of brain-derived estrogen (BDE2) on hippocampal neurogenesis with aging in female rats. 

An increasing number of studies have demonstrated that neurogenesis continuously occurs across the lifespan including rodents and humans, while the rates dramatically decline with aging [28,54,55]. However, a recent study reveals that hippocampal neurogenesis drops sharply after the first year of life and to an undetectable level in adults [28]. To determine the effects of BDE2 in hippocampal neurogenesis with aging, we first detected the cell differentiation and neurogenesis in the hippocampal DG of 1, 3, 6, 14, and 18 Mon female rats. The results revealed that cell differentiation was significantly declined over the middle age (14 Mon and 18 Mon), while newly born immature neurons clustered in SGZ area with longer dendrites through the GCL to the molecular layer in 1-Mon rats, then immature neurons sharply dropped after born 1 Mon, which consistent with the study from Sorrells [28]. However, adult rats (3 Mon and 6 Mon) still displayed a lot independent DCX+ (indicating immature neuron) cell bodies with long synaptic fibers processed in SGZ and GCL areas and a few co-labeled cells of DCX+ with NeuN+ (indicating mature neuron), which seemingly inconsistent with the findings of Sorrells et al. [28]. In elder rats (14, 18 Mon), a large number of synaptic fibers could be observed in the hilus, which might be mossy fibers projected from the DG newborn neuronal axon to the CA3 region. Increasing evidence reveals that aberrant neurogenesis, for example, abnormal elevation of neurogenesis and aberrant hippocampal circuitry remodeling lead to long-term memory impairment [56,57]. Therefore, further research is needed to determine whether, for postmenopause or old females, the unordered axons of newly born neurons could integrate into existing neural circuits or as the physical barrier leading to cognitive decline. 

Importantly, we currently revealed that BDE2 might be a crucial mediator of hippocampal neurogenesis in females with aging. We draw this conclusion based on the following facts: (1) The number of DCX+ cells in the hippocampal DG region of rats aged 1, 6, or 18 Mon KO was significantly reduced compared to age-matched WT rats; (2) In 1-Mon rats, aromatase deletion in forebrain neurons led to a significant decrease in the ratio of neurogenesis, as evidenced by the decreased percentage of DCX+/SOX2+ cells ratio to SOX2+ cells, compared with WT controls; (3) We surprisingly found that E2 immunofluorescence staining was strongly displayed in the SGZ area of 1 Mon WT rats and perfectly co-localized with DCX+ cells, while E2 was almost undetectable in the 1-Mon KO rats. These provide us with evidence that the neuronal aromatase-E2 pathway is involved in neurogenesis in female rats. Aromatase is a key enzyme of estrogen biosynthesis, which is widely expressed in neurons, astrocytes, and endothelial cells [25,40,58,59]. To further confirm the key roles of BDE2 in neurogenesis, we treated the 1-Mon female rats with letrozole, a specific inhibitor of aromatase, to inhibit total aromatase activity. As expected, we observed fewer DCX+ cells in the hippocampal SGZ area of letrozole-treated rats and newly born neurons with shorter dendrites than that in the control rats. To our acknowledgment, neurogenesis in the hippocampus mainly occurs in the development period and after birth. For rats, it takes 4 weeks for newborn neurons to express mature neuron markers [60]. Newborn neurons of 3–4 weeks are crucial to the formation of hippocampus-dependent spatial memory [61]. Further research has shown that damage to newborn neurons within 1–4 weeks can lead to long-term spatial memory impairment [62]. Indeed, our results demonstrated that treatment of letrozole for 5 consecutive days beginning the third week after birth significantly decreased neuroskeletal proteins neurofilament, spinophilin, PSD95 and suppressed CREB-BDNF pathway activation, compared to 1-Mon control rats. Consistent with our findings, there is also evidence demonstrating an essential role of endogenous hippocampal estrogen synthesis in the maintenance of hippocampal spine synapses [63,64]. 

Since BDE2 deletion reduces neurogenesis in the hippocampal DG region of female rats with aging and also damages hippocampal neuronal structural proteins. Therefore, it is possible that BDE2 deletion impairs long-term cognitive function. In our recent study, we confirmed that 18-Mon FBN-ARO-KO rats displayed defective learning and memory in the Barnes maze test. To eliminate interference from circulating E2, 6-Mon rats were ovariectomized 7d before the behavioral experiments. The results revealed that both 1-Mon and 6-Mon KO rats exhibited impaired spatial learning and memory, compared to age-matched WT rats. Additionally, although there was no significant difference in anxiety-like behavior between the two groups, 6-Mon KO rats had significant anxiety-like behavior, displaying more grooming times but fewer rearing times than the WT rats. Indeed, aromatase gene knockout in mice reduces cell proliferation, neurogenesis in the accessory olfactory bulb, and loss of the memory of male odors [65]. Further, the study using FBN-ARO-KO mice reveals that BDE2 is involved in hippocampal-dependent learning and memory [25]. Taken together, our current findings strongly suggest that BDE2 plays a key role in hippocampal-dependent learning and memory by regulating hippocampal neurogenesis in female rats with aging.

A great body of evidence demonstrates that like neurogenesis, adult gliogenesis such as astrogenesis, oligodendrogenesis, and microglia proliferation occur throughout the lifespan [66,67,68]. Gliogenesis not only presents in neurogenic regions of DG and SVZ but also in the parenchyma [44,69]. Alternations of both neurogenesis and gliogenesis have been revealed in a number of pathological conditions, e. g. Alzheimer’s disease and demyelinating diseases [70,71]. Aging is traditionally referred as a pathological process, with neurogenesis decline, synaptic atrophy and glia morphology hypertrophy [72]. Therefore, we detected the differentiation of astrocytes and microglia cells in female rats with natural aging. The results revealed that ratios of both astrocytes and microglia differentiation were significantly increased over the middle age, especially in postmenopausal rats (18 Mon). Intriguingly, aromatase knockout in forebrain neurons induced significant increases in the differentiation and activation of astrocytes. However, we did not observe markedly change in microglia differentiation although microglia activation was elevated in the hippocampal DG of 18-Mon KO rats. Notably, FBN-ARO-KO led to upregulation of neurotoxic A1 markers and downregulation of neuroprotective A2 markers of astrocytes in the hippocampus of 18-Mon female rats. Astrocytes are at the core of CNS micro-environment, their turnover into neurotoxic phenotype to impair neurons. They defined as two phenotypes neurotoxic A1 and neuroprotective A2, both sharing a pan-reactive astrocyte set of increased genes [48]. Glia cells are the major cause of abnormal neurogenesis. A2 astrocytes help neuronal differentiation and functional integration of newly produced neurons [73]. While, A1 astrocytes help to drive death of neurons and oligodendrocytes in neurodegenerative disorders [74]. A1 astrocytes lose the ability to promote neuronal survival, outgrowth, synaptogenesis [75]. Additionally, it is reported that activated microglia can induce neurotoxic A1 astrocytes [75,76]. Taken together, our results suggest that long-term lack of aromatase-BDE2 signaling accelerated neuro-inflammatory impairment by increased astrogenesis and A2 to A1 transform of astrocyte phenotypes, which might be the key cause of neurogenesis decline in adults.

## 5. Conclusions

In conclusions, in the hippocampal DG of female rats, neurogenesis persists throughout the life but sharply drops from 1 Mon after born. Neuronal differentiation significantly declines over the middle age, while differentiation ratios of astrocyte and microglia are increased. FBN-ARO-KO leads to decreased neurogenesis and impaired hippocampal-dependent cognitive with aging, while increases astrogenesis and neurotoxic A1 type, as well as microglia activation in 18-Mon female rats. However, two major limitations in this study are that male rats have not yet been investigated, because aromatase widely expresses in the brain of males and females. Additionally, the effects of ovariectomy itself on neuronal differentiation and neurogenesis with aging should be investigated in the future study.

## Figures and Tables

**Table 1:** Primer sequence used in the research.

Gene	Forward Primer	Reverse Primer
GAPDH	CAGTATGATTCTACCCACGG	CAGATCCACAACGGATACAT
Serping1	GGCGGAGAACACCAACCACAAG	TGGCACTCAAGTAGACGGCATTG
GBP2	CTCAGCAGCACCTGTCTACAAC	CACAAAGTTAGCAGAGTCGTTATCC
FKBP5	AGCCTGGGATATTGGGGTGTCTAC	CCAGCAGAGCCGTAAGCTATTC
C3D	CCACCACCTCCACCTGTTCTTAATG	GTTCACTCCTTCTCTGGGCTTGG
S100A10	TGAAGCAGAAGAAGTAGGC	CGAATTGGAGTTGGATGTTA
PTX3	ATTCTGCTTTGTGCTCTCTGGTCTG	GGGTCCTCGGTGGGATGAAGTC
Sphk1	CGGACGGCAACTCATGTTCTC	GCTCCTGTATTCTCATCLCCAAGTC
Tm4sf1	CTTCTGTACTGGCTGCTCTGATTGG	CACACTCCGGGCATCGCTAC

Cycle thresholds (CT) for single reactions were determined using MyiQ software (Bio-Rad, Hercules, CA, USA) and the target genes were normalized against GAPDH. The 2^−ΔΔCT^ method was used to calculate relative changes in gene expression.

## Data Availability

Please contact the corresponding author at ruimin-wang@163.com if necessary.

## Supplementary Materials

The following supporting information can be downloaded at: https://www.mdpi.com/article/10.3390/biology12060760/s1. Figure S1: FBN-ARO-KO Impaired Hippocampal Neurons and Cognitive Function; Figure S2: Letrozole Inhibited Neurogenesis and Neuroprotection in the Hippocampal DG Region of 1-Mon Female Rats.
