# Supplementary material for: Brain-Derived Estrogen Regulates Neurogenesis, Learning and Memory with Aging in Female Rats
Source: Biology (Basel). 2023 May 23;12(6):760. doi: 10.3390/biology12060760 (PMC10295457; doi:10.3390/biology12060760)

WB

Figure S1 FBN-ARO-KO Impaired Hippocampal Neurons and Cognitive Function.

Figure S1

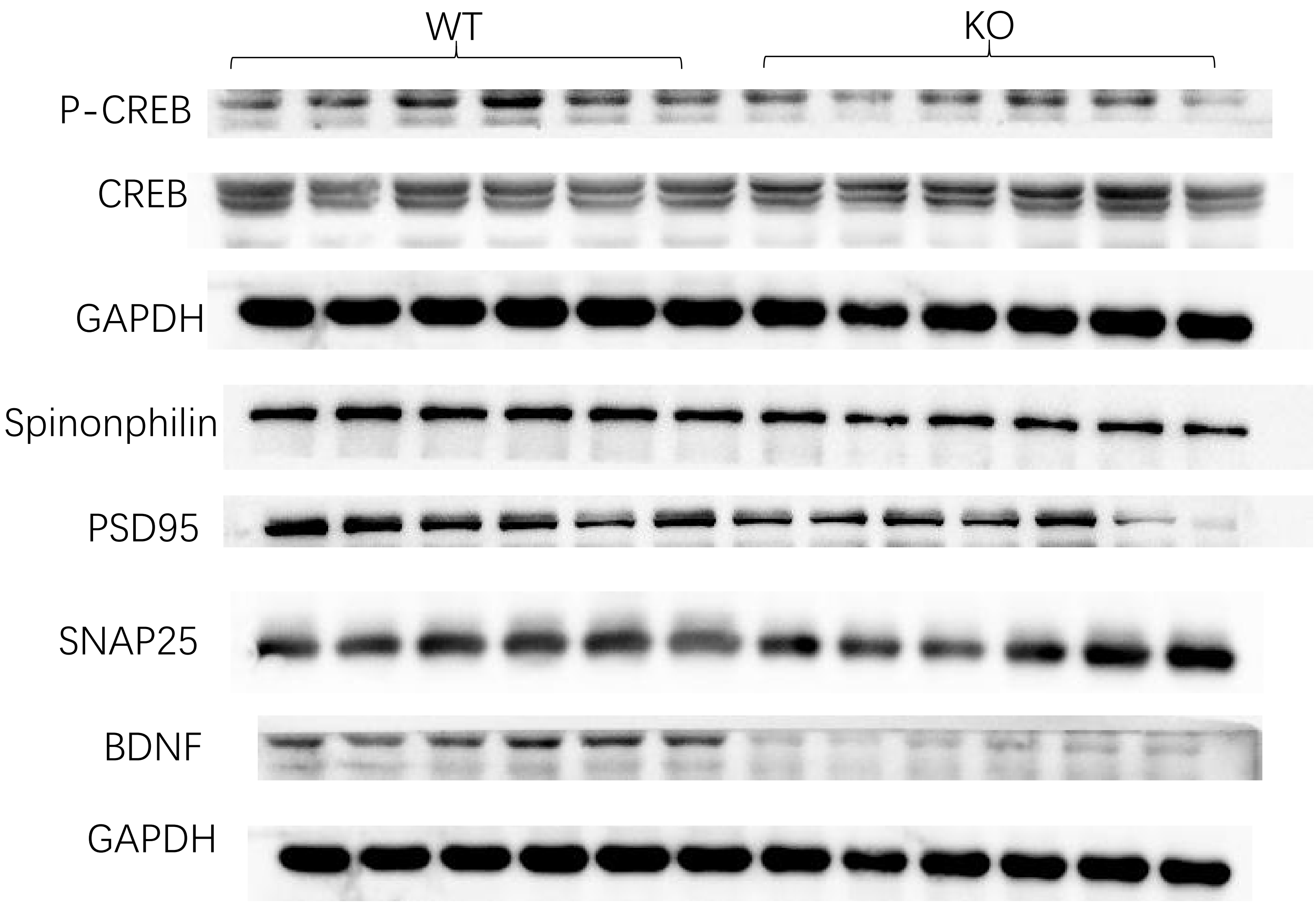

Figure S2 Letrozole Inhibited Neurogenesis and Neuroprotection in the Hippocampal DG Region of 1-Mon Female Rats.

Figure S2

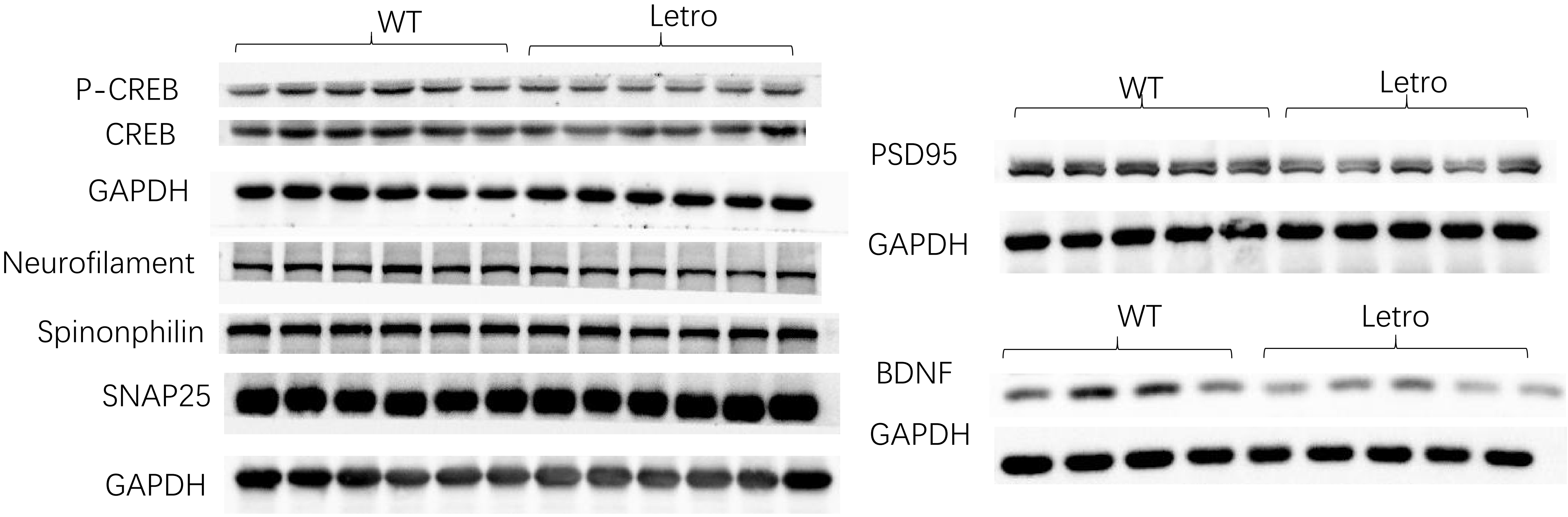

Supplement: Supplementary file 1 [file biology-12-00760-s001.zip › biology-2385949-supplementary file.pdf]
